# Kid Health Problems in Swedish Goat Herds: A Cross-Sectional Survey of Herd-Level Risk Factors and Preventive Practices

**DOI:** 10.3390/ani16050826

**Published:** 2026-03-06

**Authors:** Theodoros Ntallaris, Athina Basioura, Ioannis A. Tsakmakidis

**Affiliations:** 1 Clinical Sciences, Swedish University of Agricultural Sciences, 750 07 Uppsala, Sweden; 2 Farm Animals Clinic, School of Veterinary Medicine, Aristotle University of Thessaloniki, 54627 Thessaloniki, Greece; basioura@vet.auth.gr (A.B.); iat@vet.auth.gr (I.A.T.)

**Keywords:** goats, kid health, herd health, reproductive management, preventive management, cross-sectional study, farmer-reported data

## Abstract

Kid health problems are important welfare and productivity concerns in goat farming, yet herd-level patterns and management responses remain poorly described in many production systems. This cross-sectional study investigated farmer-reported kid health problems in Swedish goat herds and their associations with herd size and management practices. An online questionnaire distributed through national goat networks during 2024 yielded 684 completed responses, representing approximately one-third of Swedish goat keepers. Overall, 27.63% of farms (189/684) reported at least one kid health problem during the preceding three years, most commonly gastrointestinal disorders (22.8%), followed by joint-related (15.1%) and neurological conditions (9.0%). A subset of farms (6.0%) reported multiple concurrent types of kid health problems, indicating more complex herd health profiles. The proportion of farms reporting at least one kid health problem increased with herd size; large herds (>50 animals) were more likely to report health problems compared with small herds (RR = 1.51; 95% CI: 1.08–2.10), while medium-sized herds showed modest, non-significant increases. This herd-level outcome is inherently influenced by herd size, as larger herds have a higher probability of observing at least one case. Farms reporting multiple concurrent kid health problems more frequently implemented management measures such as isolation during kidding, early colostrum provision, and selenium supplementation, likely reflecting reactive adoption following previous health challenges rather than proactive prevention. Longitudinal studies using animal-level data are needed to clarify causal relationships.

## Simple Summary

Young goats are particularly vulnerable to health problems during the first weeks of life, which can raise animal welfare concerns and affect farm productivity. In this study, we surveyed 684 goat farms across Sweden to describe the occurrence of kid health problems and associated management practices at the herd level. Over one quarter of farms (27.63%) reported kid health problems during the preceding three years, most commonly gastrointestinal disorders. Farms with larger herds were more likely to report such problems; however, this comparison is influenced by herd size, as larger herds have a higher probability of observing and reporting at least one case. Several management practices, including supervision at kidding, isolation of does, selenium supplementation, and early colostrum provision, were more frequently reported by farms with multiple types of kid health problems, suggesting that these measures were often adopted in response to previous disease rather than used proactively. Network analysis further showed strong co-occurrence between pregnancy-related problems in does and subsequent health problems in kids, highlighting the close link between maternal and neonatal health. Overall, this study provides descriptive baseline information on kid health in Swedish goat herds and underlines the importance of cautious interpretation of herd-level survey data.

## 1. Introduction

The goat population in Sweden is currently expanding, although it remains a minor livestock sector, with approximately 20,000 animals managed by around 2400 holders [1]. A defining characteristic of Swedish goat husbandry is its predominantly non-commercial and multifunctional nature, with nearly 80% of holders managing fewer than ten animals [1]. Despite this growth, there is limited systematic knowledge regarding herd-level management practices and how these routines are associated with animal health and survival within the Swedish production landscape.

Neonatal mortality and morbidity represent significant challenges in goat production, with important implications for animal welfare and farm productivity [2]. Kid survival is determined by a complex interplay of maternal health during gestation, parturition outcomes, and neonatal care practices [3]. International studies have reported that larger herd sizes are often associated with higher levels of kid morbidity and mortality, a pattern that has been hypothesized to reflect increased management complexity and reduced opportunities for individual animal monitoring [4,5,6].

Preventive management practices are commonly promoted to reduce neonatal health risks. Because goat kids are born without a functional immune system, timely provision of high-quality colostrum is essential for passive transfer of immunity [7,8]. Failure of passive transfer is associated with increased susceptibility to gastrointestinal disorders, the most frequently reported health problem in young goats, as well as respiratory infections [2,9]. Maternal nutrition and trace element status, particularly selenium, are also important determinants of kid vitality and immune function, especially in regions such as Sweden where soil selenium levels are naturally low [10,11]. Understanding herd-level kid health and preventive management practices is particularly relevant in the context of regenerative and low-input livestock systems, where animal welfare, resilience, and preventive health strategies are central components.

The aim of this study was to describe the occurrence of kid health problems in Swedish goat herds and to explore herd-level associations with management practices and herd size using farmer-reported data from a wide range of production systems. By providing a national descriptive baseline, this study seeks to support the development of herd-specific management strategies and to inform future longitudinal research on preventive approaches to improve goat health and welfare in Sweden.

## 2. Materials and Methods

### 2.1. Ethical Statement

This study was based on an anonymous, voluntary questionnaire addressed to goat keepers and focused on herd-level management practices and animal health outcomes. No sensitive personal data, as defined in Article 9 of the EU General Data Protection Regulation, were collected, and no physical or psychological interventions were involved. According to the Swedish Ethics Review Act (Lag 2003:460), ethical approval is therefore not required for this type of research. All participants were informed about the purpose of the study, and submission of the questionnaire was considered to constitute informed consent. No financial incentive was offered to farmers in exchange for their participation.

### 2.2. Survey Design and Target Population

A cross-sectional survey was conducted to map management practices and health outcomes in Swedish goat herds. The target population included all Swedish goat keepers, regardless of herd size or production aim, to ensure a representative overview of the national sector. To ensure data relevance, a screening question regarding the number of goats in the herd during 2024 was included. To ensure data relevance for health outcome analyses, respondents indicating that they did not keep goats were excluded from analyses involving kid health outcomes.

The survey was developed and hosted on the web-based platform Netigate and remained open for a three-week period between March and April 2025.

### 2.3. Distribution and Recruitment

The survey was distributed using a multi-channel approach between 17 March 2025 and 11 April 2025 to maximize reach. Contact information was obtained from the Swedish Board of Agriculture’s facility register and the Swedish Goat Society’s membership records (Svenska Getförbundet). In total, 4461 email invitations were sent, of which 4272 were successfully delivered. In addition, the survey was shared in three major Swedish social media groups dedicated to goat husbandry.

The number of delivered emails exceeded the official estimate of approximately 2400 registered goat keepers in Sweden [1]. This discrepancy is likely explained by overlapping mailing lists, duplicate contacts, inactive facilities, and individuals registered at multiple holdings. Due to the use of multiple distribution channels and overlapping contact sources, a precise response rate could not be calculated.

A total of 684 completed questionnaires were received, corresponding to approximately 29% coverage of the registered goat-keeping population in Sweden.

### 2.4. Survey Structure and Content

The questionnaire consisted of 22 questions, primarily utilizing a combination of multiple-choice and open-ended text fields (Supplementary Materials). The survey was divided into key sections:

- **Herd Background** (7 questions): Herd size, location, and production focus, designed to allow comparison with previous national reports [1].
- **Kid Survival and Health** (7 questions): Data on birth rates, mortality, and the occurrence of specific diseases over the preceding three years.
- **Preventive Management** (8 questions): Implementation of specific routines such as colostrum management and parturition supervision.

The survey concluded with an open-ended comment section for respondents to provide additional context. Prior to distribution, the survey underwent pilot testing for technical functionality and clarity by veterinary students and active small ruminant keepers.

### 2.5. Definition and Categorization of Health Problems

Health problems were assessed using a predefined list of conditions included in the questionnaire. Respondents were asked to indicate which health problems they had experienced in their herd during the preceding three years by selecting all applicable options from a fixed list, with the possibility to add additional comments in free text. No clinical case definitions were provided, and responses therefore reflect farmer-reported observations rather than veterinary diagnoses.

Reported conditions were grouped into broader health categories for analysis. Kid-related health problems included gastrointestinal disorders (e.g., diarrhea, bloat, constipation), respiratory conditions (e.g., coughing, nasal discharge), neurological signs (e.g., paralysis, stiffness, circling), joint-related problems (swollen joints and/or lameness), orf (contagious ecthyma), umbilical infections, low birth weight, prematurity or malformations, stillbirths, and weakness at birth or within the first days of life. Farms could report more than one type of kid health problem, and categories were therefore non-mutually exclusive.

Pregnancy- and doe-related problems were recorded separately and included abortions, dystocia, diseases during gestation (e.g., pregnancy toxemia, vaginal prolapse), and diseases in the immediate post-parturient period (e.g., retained placenta, postpartum fever). For analytical purposes, farms reporting two or more distinct types of kid health problems were classified as having more complex herd-level health profiles.

### 2.6. Data Processing and Statistical Analysis

Descriptive statistics were calculated using SAS version 9.4 [12]. Categorical variables describing herd characteristics, management practices, and health outcomes were summarized using frequencies and percentages. For binary outcomes, 95% confidence intervals were calculated using the binomial distribution. Descriptive occurrence estimates are reported using all completed questionnaires, while analyses of specific health outcomes were conducted using complete-case data.

Associations between herd size, management practices, and kid health outcomes were evaluated using Chi-square or Fisher’s exact tests, as appropriate. The strength of associations was expressed as relative risks (RRs) with 95% confidence intervals; RR was preferred over odds ratios due to the non-rare occurrence of several outcomes.

A “high health burden” was defined as the presence of two or more concurrent kid health problems within a herd, to distinguish complex health challenges from isolated events.

Exploratory correlation analyses and network visualizations were performed in R (version 4.5.1; R Core Team, Vienna, Austria, 2020) within the RStudio platform (version 1.4.1106; RStudio, Boston, MA, USA, PBC) to illustrate interrelationships between maternal and neonatal health problems [13].

To explore co-occurrence patterns among management practices and health outcomes, a weighted undirected network was constructed using Jaccard similarity coefficients calculated from binary variable matrices. Network visualization and community detection were performed using the ‘igraph’, ‘tidygraph’, and ‘ggraph’ packages (version 1.4.1106; RStudio, Boston, MA, USA, PBC). The Louvain algorithm was applied to identify clusters of co-occurring variables. Jaccard similarity thresholds of 0.3 were used to filter weak associations and enhance network interpretability. As herd size was strongly correlated with several management practices, inclusion of multivariable models would have limited interpretability and risked over-adjustment, particularly given the descriptive aim of the study. Multivariable regression analyses were not performed due to strong collinearity among management variables and the risk of overfitting. Statistical significance was set at *p* < 0.05.

### 2.7. Handling of Missing Data

Responses were inspected for completeness prior to analysis. Missing data occurred primarily due to item non-response within otherwise completed questionnaires. Analyses were conducted using available-case analysis (pairwise deletion), such that farms with missing information for a specific variable were excluded only from analyses involving that variable, while remaining data from the same questionnaire were retained for other analyses.

As a result, denominators vary slightly between analyses and are reported explicitly where relevant in the Results and Tables. No imputation of missing values was performed, as the study was descriptive in nature and the proportion of missing data was generally low.

## 3. Results

### 3.1. Geographical Distribution

**Figure 1:**
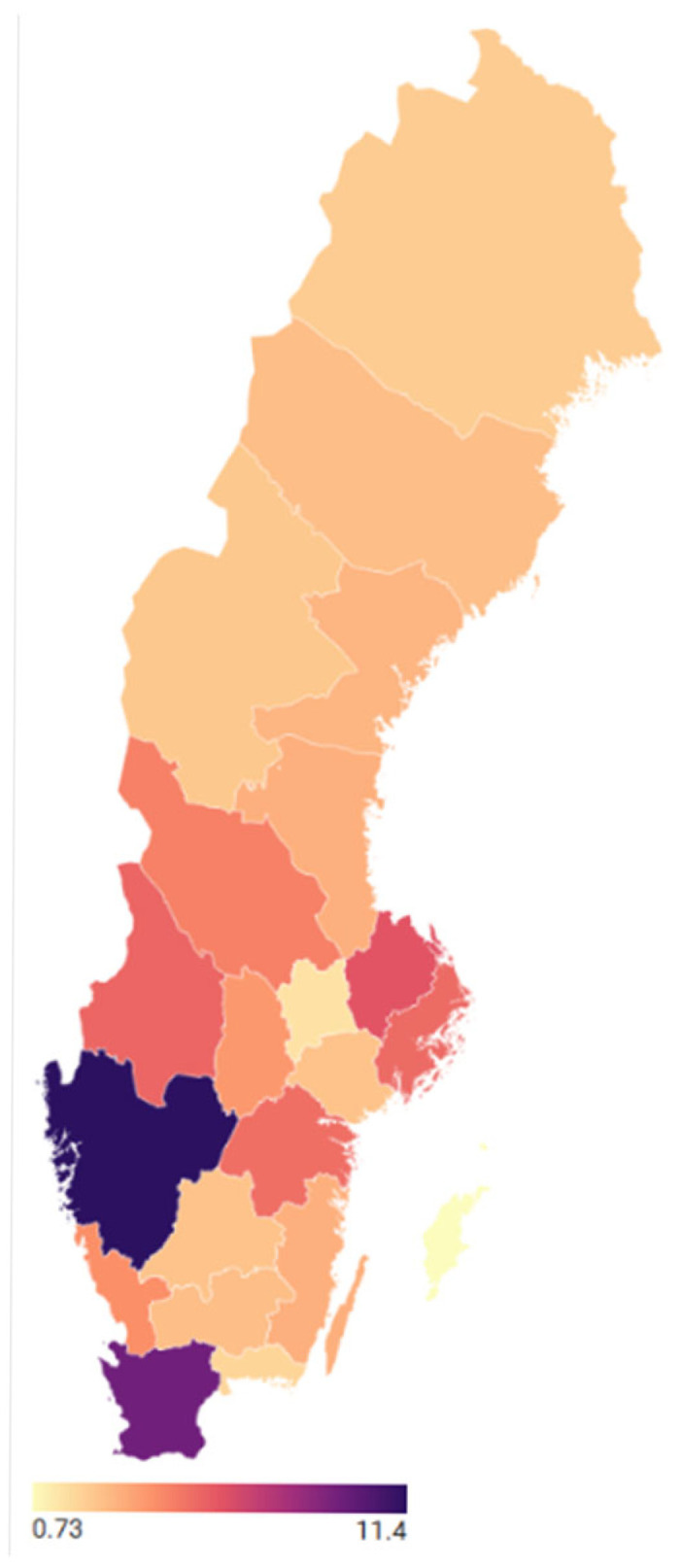
Geographical distribution of participating goat farms across Swedish counties (*n* = 684). Colour intensity represents the proportion (%) of farms per county, with darker shades indicating higher representation.

The responding goat farms were geographically distributed across all regions of Sweden (Figure 1). The highest proportions of farms were located in Västra Götaland County (11.4%), Skåne County (9.5%), and Uppsala County (6.1%). Moderate representation was observed in Östergötland, Värmland, Stockholm, and Dalarna counties, each contributing between 4% and 6% of responses. Farms from northern counties, including Norrbotten, Västerbotten, Jämtland, and Västernorrland, together accounted for approximately 10% of the study population. County information was unavailable for 14.3% of respondents.

### 3.2. The Profile of Participating Goat Farms

The participating farms represented diverse production purposes (*n* = 588; 96 farms with missing data). Multifunctional farming was the norm, with hobby farming reported by 73.47% of farms and landscape management by 59.18%. As sole purposes, hobby farming accounted for 26.36% and landscape management for 9.52%, with the most common combination being hobby with landscape management (28.40%). Pure commercial production was rare (milk alone: 0.73%; meat alone: 0.15%), reflecting the predominantly non-commercial nature of Swedish goat farming.

Herd size varied substantially (*n* = 677). Most farms maintained small herds of 1–5 animals (67.95%), followed by medium-sized herds of 6–20 animals (24.96%). Larger operations were uncommon, with 3.55% keeping 21–50 animals and only 1.62% keeping more than 50 animals. A small proportion (1.92%) reported having no animals at the time of survey.

### 3.3. The Breed Profile of the Participating Goat Farms

Several goat breeds were represented among the participating farms (*n* = 677), with most holdings reporting either a single breed or defined breed combinations. Dwarf goats were the most common, present in 34.12% of farms either as a sole breed or in combination with other breeds. Native Swedish breeds were well represented, including Swedish Landrace goats (16.99%), Jämtland goats (Jämtget; 11.37%), Göinge goats (Göingeget; 8.42%), and Lapland goats (Lappget; 5.02%).

International breeds were less common as single-breed holdings, including Boer goats (0.59%) and Angora goats (2.07%), but were more frequently kept in combination with other breeds. Mixed-breed holdings were common, often combining native Swedish breeds with dwarf goats, Boer goats, or Angora goats. Farms reporting other or unspecified breeds accounted for 3.55% of the sample. Breed information was missing for seven farms.

According to Swedish animal welfare legislation, goats must have access to pasture during the grazing season, typically between 1 May and 15 October, with minimum required grazing periods varying by county. The minimum grazing period is four months in Blekinge, Skåne, and Halland; three months in Stockholm, Uppsala, Södermanland, Östergötland, Jönköping, Kronoberg, Kalmar, Gotland, Västra Götaland, Värmland, Örebro, and Västmanland; and two months in Dalarna, Gävleborg, Västernorrland, Jämtland, Västerbotten, and Norrbotten [14].

### 3.4. Farmers’ Experience Profile

Farmer experience varied considerably (*n* = 586), with 47.27% having 0–5 years of experience, 37.71% having 5–15 years, 12.12% having 15–30 years, and only 2.90% with more than 30 years of experience. The majority of farmers across all experience levels maintained small herds of 1–5 animals (67.92% overall). Notably, newer farmers (0–5 years) were most likely to keep small herds (76.53% with 1–5 animals), while more experienced farmers showed slightly greater diversity in herd sizes. Among farmers with over 30 years of experience, herd size distribution was more balanced across small and medium categories. Larger operations (>20 animals) remained rare across all experience levels, representing only 4.95% of all farms. Ninety-eight observations had missing experience data.

### 3.5. Correlation Network Analysis of Pregnancy Problems and Kid Health Outcomes

**Figure 2:**
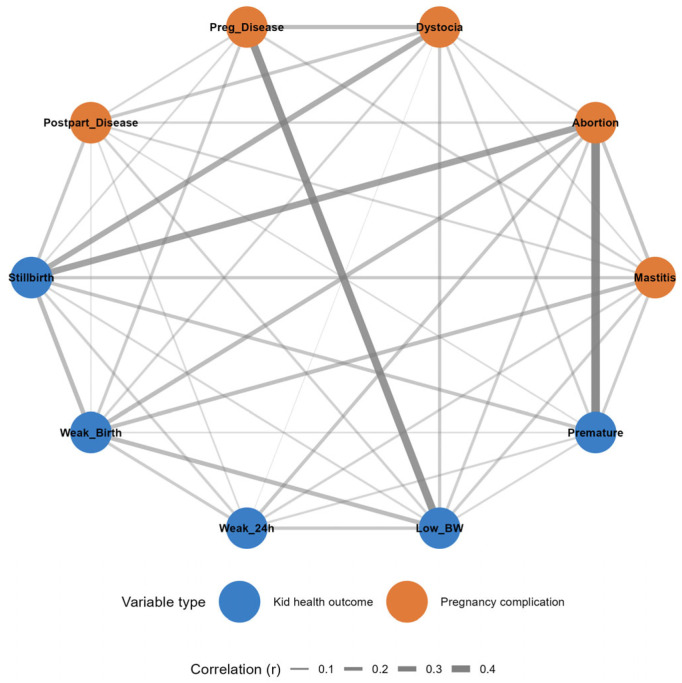
Exploratory correlation analysis of pregnancy complications and kid health outcomes in goats. Nodes represent herd-level health problems reported during the preceding three years, and edges indicate statistically significant associations between problems, with edge thickness proportional to association strength. Abbreviations used in the figure are as follows: *Weak_Birth* = weak kids at birth; *Weak_24 h* = weak kids within 24 h after birth; *Low_BW* = low birth weight; *Premature* = prematurely born kids; *Stillbirth* = full-term dead-born kids; *Mastitis* = mastitis in does; *Abortion* = abortions; *Dystocia* = parturition difficulties; *Preg_Disease* = disease during pregnancy; *Postpart_Disease* = disease after parturition.

The correlation network analysis (Figure 2) revealed complex interconnections between pregnancy complications and kid health outcomes in 684 goat farms. Network visualization identified several key patterns in the relationships among maternal and neonatal variables.

Abortion emerged as a central node in the network, showing the strongest association with premature birth (r = 0.50, *p* < 0.001), followed by notable connections with stillbirth (r = 0.37, *p* < 0.001) and weak kids at birth (r = 0.25, *p* < 0.001). Similarly, dystocia demonstrated significant associations with stillbirth (r = 0.32, *p* < 0.001) and pregnancy disease (r = 0.24, *p* < 0.001).

Pregnancy disease showed a particularly strong relationship with low birth weight (r = 0.45, *p* < 0.001). Mastitis during pregnancy exhibited moderate positive correlations with multiple kid health problems, including weak kids at birth (r = 0.23, *p* < 0.001) and stillbirth (r = 0.18, *p* < 0.001).

Among kid health outcomes, weak kids at birth were associated with low birth weight (r = 0.25, *p* < 0.001) and stillbirth (r = 0.25, *p* < 0.001), while weakness at 24 h postpartum showed connections with premature birth (r = 0.12, *p* = 0.002) and low birth weight (r = 0.18, *p* < 0.001). Notably, several pregnancy complications showed minimal or non-significant associations, such as pregnancy disease with abortion (r = −0.02, *p* = 0.69) and premature birth (r = −0.01, *p* = 0.72).

Although the variables included in the correlation network were binary, Pearson correlation coefficients were used as a pragmatic measure of association to identify patterns of co-occurrence rather than to estimate latent correlations. The objective of this analysis was exploratory visualization of interrelationships at the herd level, not inference of underlying continuous traits. Alternative measures (e.g., tetrachoric correlations) were considered but deemed less suitable given the large number of sparse binary variables and the descriptive aim of the analysis.

### 3.6. Variable Association Network and Farm Typologies

**Figure 3:**
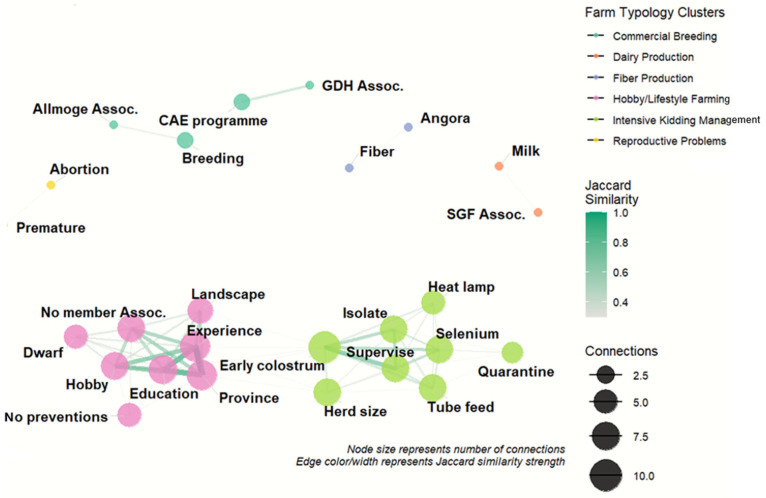
Network of associations among herd characteristics, management practices, and health outcomes in Swedish goat farms (n = 684). Nodes represent survey variables and edges indicate statistically significant associations (*p* < 0.05), with edge thickness proportional to association strength. Colors denote clusters of closely associated variables, illustrating distinct farm management and production profiles (Threshold = 0.3). Abbreviations used in the figure are as follows: *CAE programme* = participation in the caprine arthritis encephalitis control programme; *GDH Assoc.* = Gård och Djurhälsan (farm animal health service); *SGF Assoc.* = Svenska Getavelsförbundet (Swedish Goat Breeding Association); *Allmoge Assoc.* = Föreningen Allmogegeten; *Dwarf* = African dwarf goats; *Angora* = Angora goats; *Fiber* = fiber production; *Milk* = milk production; *Landscape* = landscape management; *Hobby* = hobby or lifestyle farming; *No member Assoc.* = no membership in external organizations; *No preventions* = no reported preventive management measures; *Early colostrum* = provision of colostrum within the first hours after birth; *Supervise* = supervision during kidding; *Isolate* = isolation of does around parturition; *Tube feed* = bottle or tube feeding of weak kids; *Heat lamp* = provision of supplemental heat; *Selenium* = selenium and/or vitamin E supplementation; *Quarantine* = isolation of newly purchased animals; *Province* = county of herd location; *Experience* = years of goat-keeping experience; *Education* = education or training related to animal husbandry; *Herd size* = categorized number of goats in the herd; *Abortion* = abortions; *Premature* = premature births.

Exploratory network analysis identified distinct clusters of co-occurring herd characteristics, management practices, and health outcomes (Figure 3). The largest cluster was characterized by intensive kidding-related management practices, including early colostrum provision, tube feeding, use of heat lamps, selenium supplementation, isolation and supervision during kidding, and quarantine measures. This cluster was strongly associated with larger herd sizes.

A separate cluster reflected a hobby-oriented farming profile, characterized by dwarf breeds, landscape management, smaller herd sizes, limited organizational affiliation, and lower production intensity. Additional clusters represented specialized production systems, including fiber production (Angora goats) and dairy production associated with membership in the Swedish Goat Association.

Reproductive health problems formed a distinct cluster, with abortion and premature birth showing strong co-occurrence but limited overlap with management-related variables. Overall, the network revealed heterogeneous farm typologies within the Swedish goat sector and highlighted systematic co-occurrence of management practices rather than isolated interventions.

### 3.7. Herd-Level Occurrence and Complexity of Kid Health Problems

Early colostrum administration was more common among farms without reported kid health problems (23.83%). The occurrence of at least one kid health problem differed significantly by herd size (χ^2^ = 14.9, df = 4, *p* = 0.005). A significant increasing trend was observed, with larger herds reporting kid health problems more frequently (Cochran–Armitage trend test, *p* = 0.038). Farms with more than 50 animals reported kid health problems in 63.64% of cases, compared with 24.68% of farms keeping 1–5 animals.

The proportion of farms reporting at least one kid health problem increased with herd size (Table 1). Kid health problems were reported by 24.7% of small herds, 32.0% of medium-sized herds, and 50.0% of large herds. Compared with small herds, medium-sized herds showed a non-significant increase in likelihood of herd-level reporting (RR = 1.11; 95% CI: 0.99–1.24), whereas large herds had a significantly higher likelihood of herd-level reporting kid health problems (RR = 1.51; 95% CI: 1.08–2.10). It should be noted that this outcome reflects herd-level reporting of at least one health problem rather than animal-level incidence, which inherently increases the probability of reporting in larger herds. Kid health problems were reported by the majority of farms during the preceding three years (Table 2). Gastrointestinal disorders were the most frequently reported specific condition, occurring on 14.6% (100 from 684 farms; 95% CI: 12.1–17.5). Joint-related problems were reported by 8.6% of farms (59 from 684 farms; 95% CI: 6.7–11.0), while respiratory disorders were reported by 5.0% (34 from 684 farms; 95% CL: 3.5–6.9). Neurology problems (4.8%; 33 from 684 farms; 95% CL: 3.5–6.8) and orf (2.3%; 16 from 684 farms; 95% CI: 1.4–3.9) were reported less frequently. Gastrointestinal disorders were reported significantly more frequently than the second most common condition, joint disorders (χ^2^ = 11.4, df = 1, *p* = 0.0007). In total, 189 farms reported at least one health problem corresponding to 27.63% of the farms (n = 684). However, 56 farms activily ticked “no health problems” in the questionnaire, corresponding to 8.2% of the farms (n = 684).

The occurrence and complexity of perceived kid health problems differed significantly across herd size categories (Table 3). The proportion of farms reporting no kid health problems decreased with increasing herd size, from 75.3% in small herds to 68.0% in medium-sized herds and 50.0% in large herds.

Conversely, the proportion of farms reporting multiple kid health problems increased markedly with herd size, affecting 4.1% of small herds, 7.1% of medium-sized herds, and 27.8% of large herds. Overall, herd size was strongly associated with the number of reported kid health problems (χ^2^ = 35.3, *p* < 0.0001).

Associations between herd characteristics, management practices, and the likelihood of reporting a high burden of kid health problems (defined as ≥2 reported problem categories) are summarized in Table 4. Farms reporting a high burden of kid health problems were more likely to have larger herd sizes, with the highest occurrence observed in herds exceeding 50 animals. Several management practices were also more commonly reported among farms with a high health burden, including isolation of does during kidding, increased supervision at parturition, early colostrum provision, and selenium supplementation. In contrast, housing system, production orientation, and most routine preventive measures did not differ substantially between farms reporting a high versus low burden of kid health problems. These associations should be interpreted at the herd level and reflect reported management responses rather than causal risk factors.

## 4. Discussion

### 4.1. Herd Size as a Marker of Management Complexity

Kid health problems were more frequently reported in larger herds, with large operations showing a significantly higher probability of reporting compared with small herds. Similar associations between herd size and kid morbidity or mortality have been reported in European dairy goat systems [4,5,6]. The wide variation in herd sizes observed in the present study likely reflects the heterogeneous nature of Swedish goat keeping, where the majority of holders manage small hobby herds with limited formal training in animal husbandry. As shown in Section 3.4, nearly half of all farmers (47.27%) had fewer than five years of experience, and most maintained herds of 1–5 animals. This pattern suggests that many goat keepers may have limited exposure to herd health management challenges that become more apparent as herd size increases. Inexperience and limited specialist knowledge may reduce the likelihood of recognising, preventing, or reporting health problems in smaller herds, while larger operations may benefit from more systematic monitoring despite their greater complexity. These findings highlight the importance of targeted educational initiatives and improved access to veterinary advisory services for goat keepers at all experience levels, particularly given the expanding nature of the Swedish goat sector.

Accordingly, herd size should not be interpreted as a direct causal risk factor for poor kid health, but rather as a contextual indicator associated with increased management complexity (associated herd-level characteristic). Larger herds may face challenges related to hygiene, disease pressure, and coordination of perinatal care, while also potentially benefiting from more systematic observation and record-keeping, which could increase the likelihood of problem detection and reporting. As no data on labor input or monitoring intensity were collected in this study, reduced individual animal monitoring should be regarded as a plausible hypothesis rather than a demonstrated mechanism [15,16].

Importantly, kid health problems were not evenly distributed across herds but clustered within a subset of farms, suggesting that herd-specific management practices and prior disease experience may be more influential than herd size alone [17,18]. Given the cross-sectional design and reliance on farmer-reported data, the observed associations should be interpreted as descriptive and exploratory rather than causal [19,20].

### 4.2. Preventive Practices: Proactive Versus Reactive Implementation

The relationship between preventive management practices and kid health outcomes revealed an important temporal paradox. Farms reporting multiple concurrent kid health problems were more likely to report the use of several preventive measures, including isolation during kidding, supervision of parturition, and selenium supplementation. In contrast, early colostrum provision, a practice with well-established protective effects on neonatal immunity [10,21], was more commonly reported among farms that did not report kid health problems.

This pattern is consistent with reactive rather than proactive implementation of preventive strategies. Farms experiencing repeated or complex health challenges may intensify management interventions in response to prior disease events, a phenomenon described in other livestock production systems [22,23]. Conversely, early colostrum provision may reflect a foundational management practice implemented routinely before health problems occur, thereby reducing the likelihood of disease establishment rather than responding to it.

Due to the cross-sectional design, the temporal sequence between management practices and health outcomes cannot be determined. It is therefore not possible to infer whether preventive measures were implemented before or after the occurrence of kid health problems. The observed associations should be interpreted as indicators of farm management history and response patterns rather than evidence of protective or adverse causal effects.

Taken together, the findings suggest the presence of differing management profiles among farms, characterized by variation in the timing and intensity of preventive practices. These results underscore the importance of distinguishing between proactive baseline management routines and reactive interventions adopted following disease experience when interpreting herd-level associations in cross-sectional surveys.

Among the preventive practices recorded in this study, colostrum provision, selenium supplementation, and use of heat lamps merit particular attention. Early colostrum provision was the most commonly reported measure (37.7% of farms) and the practice most consistently associated with absence of kid health problems. Kids are born agammaglobulinaemic and entirely dependent on immunoglobulin absorption during the first hours of life, and failure of passive transfer is strongly associated with increased susceptibility to gastrointestinal and respiratory disease [2,7,9]. Tube feeding, reported by 20.3% of farms, represents an important complementary measure when suckling is insufficient or delayed [6]. Selenium supplementation, reported by 25.1% of farms, is particularly relevant in Sweden where soil selenium levels are naturally low, and deficiency during late pregnancy has been associated with weak kids, reduced colostrum quality, and increased neonatal mortality [10,11]. Its more frequent use among high health burden farms suggests reactive rather than proactive adoption, despite strong evidence supporting routine supplementation of pregnant does. Finally, heat lamp use was reported by only 17.7% of farms, which is low given the Swedish climate. Newborn kids have limited thermoregulatory capacity and are highly vulnerable to hypothermia in the immediate neonatal period, particularly when born weak or premature [2,24]. Wider adoption of thermal support in kidding areas represents a practical, low-cost improvement with direct relevance to neonatal survival.

### 4.3. Pregnancy-Related Problems and Maternal–Neonatal Health Linkages

Pregnancy-related complications were reported by a minority of farms, indicating generally good reproductive health at the herd level during the study period. However, consistent with observations from European dairy goat systems [4,5], these problems were not evenly distributed but instead clustered within a subset of herds, suggesting the presence of herd-specific profiles with higher likelihood of herd-level reporting.

The network analysis demonstrated that pregnancy complications tended to co-occur and were closely linked to subsequent kid health problems. In particular, abortion and dystocia functioned as central connecting variables between maternal health disturbances during gestation and adverse neonatal outcomes. These associations are biologically plausible and align with existing evidence that maternal metabolic, nutritional, or infectious challenges during pregnancy can compromise fetal development, birth outcomes, and neonatal vigor [2,3,25]. However, the network structure reflects patterns of co-occurrence rather than causal pathways.

The relatively low overall frequency of reported pregnancy-related problems in this study compared with some intensive commercial systems may be influenced by differences in herd structure and production context. Swedish goat herds are predominantly small to medium-sized and often managed for multiple purposes, which may allow closer observation of individual animals and timely intervention around parturition. While this interpretation is consistent with previous observations in small-scale ruminant systems [26,27], direct measures of monitoring intensity were not available and this explanation should therefore be considered a hypothesis rather than a confirmed mechanism.

Overall, these findings reinforce the concept that kid health is closely linked to maternal health during gestation and that reproductive and neonatal outcomes should be considered as part of an integrated maternal–neonatal health system. Interventions aimed at improving kid health may therefore benefit from addressing prenatal associated herd-level characteristics alongside postnatal management practices.

### 4.4. Methodological Considerations and Study Limitations

Several methodological limitations should be considered when interpreting these findings. First, the cross-sectional study design excludes causal interpretation. The observed associations may reflect reverse causation, such as management practices adopted in response to prior disease events, or residual confounding by unmeasured herd-level factors. Longitudinal studies are required to establish temporal relationships and assess causal pathways between management practices and kid health outcomes.

Second, all health outcomes were based on farmer self-report rather than veterinary diagnoses or standardized clinical records. This introduces the potential for recall bias, social desirability bias, and heterogeneity in outcome definitions across farms. Differences in farmer experience, production objectives, and record-keeping practices may influence the recognition and reporting of health problems, particularly between small hobby farms and larger commercial herds [19,20].

Third, health outcomes were assessed at the herd level using a binary indicator of the presence of at least one reported health problem, rather than as animal-level incidence rates. As a result, larger herds are mathematically more likely to report at least one case simply due to greater numbers of animals at risk, independent of underlying disease risk. This inherent probability bias limits direct comparison across herd sizes and with studies reporting animal-level morbidity or mortality rates [5,28]. The use of herd-level measures was necessary given the predominantly small-scale and multifunctional structure of Swedish goat farming, where detailed individual-animal records are less consistently available [27].

The predominantly small size of Swedish goat herds should be considered when interpreting the findings and comparing them with studies from other countries. Even herds classified as “large” in the present study remain small relative to commercial dairy goat operations in many European production systems. This structural difference may influence management practices, disease dynamics, and reporting patterns, and limits direct comparability with studies conducted in more intensive or specialized goat industries. Consequently, the results are most applicable to small-scale and multifunctional goat production systems, which nevertheless represent a substantial proportion of goat farming globally.

The findings of this study have several practical implications for goat health management in Sweden. Larger farms, which reported higher burdens of kid health problems, also more frequently implemented structured management protocols such as isolation at kidding, supervision during parturition, selenium supplementation, and early colostrum provision. While these practices are well-established components of neonatal care in commercial goat systems [2,6,21], their reactive rather than proactive adoption, as suggested by the cross-sectional associations observed here, limits their protective potential. In contrast, vaccination programmes were remarkably uncommon across all farm types, with 61.7% of farms reporting no vaccination at all, which may reflect limited veterinary engagement, low perceived disease risk, or insufficient awareness of available preventive options in the Swedish goat sector.

For small-herd farmers, who represent the majority of Swedish goat keepers and often have limited formal training, the priority areas for improvement are likely to include basic neonatal management routines such as ensuring timely and adequate colostrum provision, navel disinfection, and monitoring of kids during the first 24 h of life. These practices are low-cost, evidence-based, and particularly relevant for reducing gastrointestinal disorders, the most commonly reported health problem in this study. For farms experiencing a high burden of concurrent health problems, a more systematic approach is warranted, including structured herd health planning in collaboration with a veterinarian, regular review of nutritional management during late pregnancy, particularly selenium and energy supplementation, and implementation of biosecurity measures such as quarantine of newly introduced animals.

Overall, these findings suggest that improving kid health outcomes in Swedish goat herds requires a differentiated approach tailored to farm size, experience level, and existing health challenges. National awareness campaigns, accessible veterinary advisory services, and practical guidelines developed specifically for small-scale and hobby goat keepers could play an important role in translating existing scientific knowledge into routine farm practice. Future longitudinal studies with animal-level data are needed to evaluate the effectiveness of specific interventions and to identify the most impactful points of intervention across different production contexts.

Finally, the network correlation analysis identifies patterns of co-occurrence among management practices and health outcomes but cannot determine the direction of associations or distinguish direct from indirect relationships. Despite these limitations, the overall consistency of the findings with existing literature across different production systems supports their robustness and relevance as descriptive baseline data.

## 5. Conclusions

This cross-sectional survey provides the first nationwide, herd-level description of kid health problems in Swedish goat farms. Gastrointestinal disorders were the most frequently reported condition, and both kid and pregnancy-related health problems clustered within a subset of herds, indicating substantial between-farm heterogeneity. Larger herds were more likely to report kid health problems, though this reflects herd-level measurement probability rather than causal risk. Preventive management practices were more commonly reported by farms with higher health burdens, suggesting reactive rather than proactive implementation. These findings underline the need for targeted management guidance for small-scale goat keepers and longitudinal studies with animal-level data to clarify causal relationships and evaluate the effectiveness of preventive interventions.

## Figures and Tables

**Table 1:** Association between herd size category and the occurrence of kid health problems on Swedish goat farms.

95% CI	Relative Risk (RR) ^1^	Farms with ≥1 Kid Health Problem, n (%)	Farms (n)	Herd Size Category
–	Reference	114 (24.7)	462	Small (1–20 goats)
0.99–1.24	1.11	54 (32.0)	169	Medium (21–50 goats)
1.08–2.10	1.51	18 (50.0)	36	Large (>50 goats)

^1^ Relative risk (RR) was calculated using small herds as the reference category (RR = 1.00 by definition). RR values for medium and large herds represent the likelihood of reporting at least one kid health problem relative to small herds. Kid health problems were defined as the occurrence of at least one reported gastrointestinal, respiratory, neurological, joint, or orf-related health problem during the previous three years.

**Table 2:** Frequency of farmer-reported kid health problems, reproductive/neonatal problems, and preventive management practices across Swedish goat farms (*n* = 684).

Category	Variable	n	%
Kid health problems ^1^	Gastrointestinal disorders	100	14.6
	Respiratory disorders	34	5.0
	Neurological disorders	33	4.8
	Joint disorders	59	8.6
	Orf (contagious ecthyma)	16	2.3
Reproductive/neonatal problems ^2^	Mastitis during pregnancy	42	6.1
	Dystocia	35	5.1
	Abortion	28	4.1
	Weak kids at birth	32	4.7
	Weak kids at 24 h	20	2.9
	Premature kids	20	2.9
	Low birth weight	10	1.5
	Pregnancy disease	3	0.4
Preventive management practices ^3^	Early colostrum provision	258	37.7
	Supervision at kidding	217	31.7
	Isolation at kidding	180	26.3
	Selenium supplementation	172	25.1
	Tube feeding of kids	139	20.3
	Heat lamp use	121	17.7
	CAE control programme	87	12.7
	Quarantine of new animals	82	12.0
	Group housing during pregnancy	38	5.6
	Navel disinfection	23	3.4
	Colostrum quality check	14	2.0

^1^ Percentages calculated as proportion of all responding farms (n = 684). Farms could report more than one health problem category; categories are therefore not mutually exclusive, except “no health problems reported”. ^2^ Percentages calculated as proportion of all responding farms (n = 684). Farms could report more than one reproductive/neonatal problem; categories are not mutually exclusive. ^3^ Percentages calculated as proportion of all responding farms (n = 684). Farms could report more than one management practice; categories are not mutually exclusive.

**Table 3:** Distribution of kid health problems by herd size category ^1^.

Total	Multiple Problems n (%)	Single Problem n (%)	No Problems n (%)	Herd Size Category
462	19 (4.1)	95 (20.6)	348 (75.3)	Small (<20 goats)
169	12 (7.1)	42 (24.9)	115 (68.0)	Medium (21–50 goats)
36	10 (27.8)	8 (22.2)	18 (50.0)	Large (>50 goats)
667	41 (6.1)	145 (21.7)	481 (72.1)	**Total (excl. missing ^2^)**

^1^ Percentages are calculated using farms with complete responses for each health category; denominators therefore vary slightly between variables. ^2^ Seventeen farms with missing herd size data excluded from herd size stratification.

**Table 4:** Association between herd characteristics, management practices, and farms reporting a high burden ^1^ of kid health problems.

*p*-Value	High Burden (%)	Low Burden (%)	Category	Variable
<0.0001	4.1	95.9	Small	**Herd size ^2^**
	7.1	92.9	Medium	
	27.8	72.2	Large	
0.0008	4.2	95.8	No	**Isolation during kidding**
	11.1	88.9	Yes	
0.0005	3.9	96.2	No	**Supervision during kidding**
	10.6	89.4	Yes	
0.012	4.2	95.8	No	**Early colostrum provision**
	8.9	91.1	Yes	
<0.0001	3.9	96.1	No	**Selenium supplementation**
	12.2	87.8	Yes	

^1^ High burden defined as farms reporting ≥2 kid health problems during the study period. ^2^ Herd size categories were defined as small (1–20 goats), medium (21–50 goats), and large (>50 goats), based on the total number of animals reported per farm.

## Data Availability

The raw data supporting the conclusions of this article will be made available by the authors on request. The original data presented in the study are openly available in the SLU repository: https://stud.epsilon.slu.se/, accessed on 20 February 2026.

## Supplementary Materials

The following supporting information can be downloaded at: https://www.mdpi.com/article/10.3390/ani16050826/s1. File S1: Questionnaire translated version from Swedish.

## References

[1] Swedish Board of Agriculture (2019). Statistikrapport 2019:01—Goat Holdings and Goat Breeders in Sweden.

[2] Dwyer C.M., Conington J., Corbiere F., Holmøy I.H., Muri K., Nowak R., Rooke J., Vipond J., Gautier J.M. (2016). Invited review: Improving neonatal survival in small ruminants: Science into practice. Animal.

[3] Robertson S.M., Atkinson T., Friend M.A., Allworth M.B., Refshauge G. (2020). Reproductive performance in goats and causes of perinatal mortality: A review. Anim. Prod. Sci..

[4] Anzuino K., Bell N.J., Bazeley K.J., Nicol C.J. (2010). Assessment of welfare on 24 commercial UK dairy goat farms based on direct observations. Vet. Rec..

[5] Balasopoulou V., Kalić M., Zablotski Y., Zerbe H., Voigt K. (2022). Management und Kitzsterblichkeit in süddeutschen Milchziegenbetrieben [Management practices and kid mortality in southern German dairy goat herds]. Berl. Münchener Tierärztliche Wochenschr..

[6] Bélanger-Naud S., Cinq-Mars D., Julien C., Arsenault J., Buczinski S., Lévesque J., Vasseur E. (2021). A survey of dairy goat kid-rearing practices on Canadian farms and their associations with self-reported farm performance. J. Dairy Sci..

[7] Argüello A., Castro N., Zamorano M.J., Castro-Alonso A., Capote J. (2004). Passive transfer of immunity in kid goats fed refrigerated and frozen goat colostrum and commercial sheep colostrum. Small Rumin. Res..

[8] Smith M.C. (2009). Goat Medicine.

[9] O’Brien J.P., Sherman D.M. (1993). Serum immunoglobulin concentrations of newborn goat kids before and after consumption of colostrum. Am. J. Vet. Res..

[10] Kachuee R., Moeini M., Souri M. (2013). Effects of organic and inorganic selenium supplementation during late pregnancy on colostrum and serum Se status, performance and passive immunity in Merghoz goats. Anim. Reprod. Sci..

[11] Sjödin E. (2024). Getter.

[12] SAS Institute Inc (2013). SAS Software.

[13] R Foundation for Statistical Computing R: A Language and Environment for Statistical Computing. https://www.R-project.org/.

[14] Swedish Board of Agriculture (2019). Statens Jordbruksverks Föreskrifter Och Allmänna Råd Om Hållande Av Getter I Jordbruket [Regulations and General Advice on the Keeping of Goats in Agriculture].

[15] Hempstead M.N., Lindquist T.M., Shearer J.K., Shearer L.C., Plummer P.J. (2021). Health and welfare survey of 30 dairy goat farms in the Midwestern United States. Animals.

[16] Hempstead M.N., Lindquist T.M., Shearer J.K., Shearer L.C., Cave V.M., Plummer P.J. (2021). Welfare assessment of 30 dairy goat farms in the Midwestern United States. Front. Vet. Sci..

[17] Anzuino K., Knowles T.G., Lee M.R.F., Grogono-Thomas R. (2019). Survey of husbandry and health on UK commercial dairy goat farms. Vet. Rec..

[18] Kalić M. (2021). Kitzgesundheit in Süddeutschen Milchziegenherden—Fragebogengestützte Bestandsuntersuchungen. Ph.D. Thesis.

[19] Phythian C., Phillips K., Wright N., Morgan M. (2014). Sheep health, welfare and production planning. 1. Recording and benchmarking performance indicators of flock health and production. InPractice.

[20] Wong J.T., Vance C.J., Peters A.R. (2021). Refining livestock mortality indicators: A systematic review. Gates Open Res..

[21] Bélanger-Naud S., Vasseur E. (2021). Graduate Student Literature Review: Current recommendations and scientific knowledge on dairy goat kid rearing practices in intensive production systems in Canada, the United States, and France. J. Dairy Sci..

[22] Ratsep E. (2020). Kid Mortality on Ontario Goat Farms. Master’s Thesis.

[23] Slayi M., Maphosa V., Fayemi O.P., Mapfumo L. (2014). Farmers’ perceptions of goat kid mortality under communal farming in Eastern Cape, South Africa. Trop. Anim. Health Prod..

[24] Holmøy I.H., Waage S., Granquist E.G., L’Abee-Lund T.M., Ersdal C., Hektoen L., Sørby R. (2017). Early neonatal lamb mortality: Postmortem findings. Animal.

[25] Swarnkar C.P., Sonawane G.G., Sharma S.R. (2021). Best Practices for Reducing the Neonatal Mortality in Sheep Flocks.

[26] Ganter M., Benesch C., Bürstel D., Ennen S., Kaulfuss K.-H., Mayer K., Moog U., Moors E., Seelig B., Spengler D. (2012). Empfehlungen für die Haltung von Schafen und Ziegen der Deutschen Gesellschaft für die Krankheiten der kleinen Wiederkäuer, Fachgruppe der DVG. Teil 1. Tierärztliche Prax. Großtiere.

[27] Manek G., Simantke C., Sporkmann K., Georg H., Kern A. (2017). Systemanalyse der Schaf- und Ziegenmilchproduktion in Deutschland. Proceedings of the 14. Wissenschaftstagung Ökologischer Landbau, Freising-Weihenstephan, Germany, 7–10 March 2017.

[28] Binns S.H., Cox I.J., Rizvi S., Green L.E. (2002). Risk factors for lamb mortality on UK sheep farms. Prev. Vet. Med..

