# Supplementary material for: Kid Health Problems in Swedish Goat Herds: A Cross-Sectional Survey of Herd-Level Risk Factors and Preventive Practices
Source: Animals (Basel). 2026 Mar 6;16(5):826. doi: 10.3390/ani16050826 (PMC12984889; doi:10.3390/ani16050826)
Supplement: Supplementary file 1 [file animals-16-00826-s001.zip › animals-4135727-supplementary.pdf]

File S1 (Translated version from Swedish)

General information about the herd 7 questions

1. How many goats (adult females, bucks and kids) did you have in your herd in 2024? (only one answer possible)

O 1-5
O 5-20
O 20-50
O 50+
O No

2. What breed(s) are your goats? Please select all that apply.

- Swedish goat breed
- Jämtget
- Lapp goat
- Goeget
- African dwarf goat
- Boer
- Angora
- Please specify other (free text)___
-

3. How many years of experience do you have in keeping goats? (Only one answer possible)

- 0-5 years
- 5-15 years
- 15-30 years
- More than 30 years

4. What education do you have in goat husbandry specifically, and/or other animal knowledge? (for example, natural farming high school, lambing course...)

Free text:_______

5. In which region is your goat farm located?

(Scroll list of Swedish Province)

6. What is your main purpose for keeping goats? (Select all that apply)

- Milk for dairy/own dairy
- Meat for sale
- Meat/milk for own consumption
- Social/hobby
- Landscape maintenance
- Visiting activities
-

- Breeding/livestock sales (including breed preservation)
- Wool/fur/fibers,
- Please specify otherwise (free text)___

7. Are you affiliated with any external organization or other action plan? (Select all that apply)

- CAE control program, enter status (free text)____
- Farm and animal health
- Conditional use of medicines Swedish
- Goat Breeders' Association The
- Allmogegeten Association
- Other association or organization? Please specify (free text)_

Horns and dehorning 7 questions

I

8. Do you have your goats dehorned? f yes, at what age does this most often occur?

- 0–7 days
- 1–2 weeks
- 2–4 weeks
- After 4 weeks of age, do
- not dehorn

9. Do you have your bucks castrated? Choose the option that best suits you

- Yes, most of the time
- Sometimes
- No, usually not.

10. Do you vaccinate your goats? Select all the options that are correct.

O Yes,

- annually
- during pregnancy
- all the kids
- before dehorning
- before castration
- If necessary (e.g. disease outbreak, or prior to sale), please state reason___

O No, never

O If YES, please state which vaccine/against which diseases ____

11. Have you ever encountered these problems because of the horns or because of dehorning?

Problems due to the horns (for example, bleeding horn injuries/broken horns, suspected pole injuries, stuck in furnishings/fences, etc.). Select the option that best fits

- Yes, recovered on its own or only required nursing care
- Yes, required veterinary care
- Yes, resulted in exit from the herd (suicide, euthanasia or slaughter)
- No, never
- Not relevant (have not had horned goats)

Problems after dehorning (for example, insufficient dehorning/horn growth after dehorning, or nasal discharge/infection/fever after dehorning)

- Yes, recovered on its own or only required nursing care Yes,
- required veterinary care
- Yes, resulted in exit from the herd (suicide, euthanasia or slaughter)
- No, never
- Not relevant (have not had dehorned goats)

Do you have any comments on problems you have experienced due to horns or after dehorning: (free text)______

12. Do you use other problem-solving measures to prevent injuries due to horns? Select all that apply.

- No,
  - O not relevant (the animals are dehorned or lack horns)
  - O no problems with the horns have been experienced
  - O not yet but have been thinking about doing
- Yes,
  - O files/rounds the tips of the horns
  - O uses protection on the horns/tips
  - O separates the animals so that the groups are calm
  - O uses furnishings so that everyone can take cover and eat

13. What do you think are the main arguments for dehorning goats?

- Easier handling (including use of furnishings)
- Calmer animal group
- Less damage to animals
- More milk production

- To prevent animals from getting caught by the horns
- Other, please specify (free text)___

14. What do you think are the main reasons for not dehorning goats?

- Experience no need or problem with the horns.
- Cost
- Buy dehorned animals or have naturally hornless/horned animals
- Want to avoid the risk of complications from dehorning
- Experience reluctance to have dehorned by the veterinarian
- Other, please specify (free text)___

Goat kid survival

15. Which of these health problems and illnesses have you experienced in the crew in the last 3 years?
Please tick all that apply.

- Stomach/intestinal problems (such as dysentery, diarrhea, or constipation)
- Respiratory symptoms (e.g. cough, runny nose)
- Neurological symptoms (e.g. paralysis, stiffness, circling gait)
- ORF (scabs/blisters on mouth/nose/udder)
- Swollen joints and/or lameness

- Mastitis/mastitis
- Abortions/ involuntarily empty goats
- Difficulties in birth
- Illness during pregnancy (for example, vaginal prolapse, preeclampsia, etc.)
- Postpartum illness (for example, retained placenta, fever, etc.)

- Stillbirths (full-term but dead fetus)
- Malformed or premature kids with abnormally low birth weight
- Weak kids at birth (cannot stand or eat without help)
- Weak kids 1-2 days after birth
- Belly button infections

Other diseases and problems, or other comments on the above?
Free text:______________

16. How many kids have survived to slaughter/sale/breeding in your herd in the last 3 years? (Include both born and purchased kids) (numbers only)____

Total number of kids born (not purchased): (numbers only)____
Total number of kids lost (dead or euthanized, not sold or slaughtered): (numbers only)____

17. What preventive measures for pregnant goats and newborn kids do you use in your herd?
Mark all that apply.

- Ensures that the kittens receive colostrum within the first hours after birth
- Checks colostrum quality
- Bottle or tube feeds weak kids
- Dips/sprays umbilical cord with antiseptic
- Uses heat lamp/heated space for kids
-
- Grouping pregnant animals based on nutritional needs
- Provide pregnant animals and/or newborn kids with selenium/vitamin E supplements
- Separates the goat from the group before or after giving birth
- Monitors and assists during giving birth if necessary
- Isolate purchased animals (quarantine)
- Not relevant (does not have pregnant animals/does not have kids)

Other preventive measures you use or comments on what you choose to use/ not use: (free text)_______

18. Have you encountered any particular challenges in improving the survival of your goat kids? (free text)______
